# Epigenetics in Neurodevelopment: Emerging Role of Circular RNA

**DOI:** 10.3389/fncel.2019.00327

**Published:** 2019-07-19

**Authors:** Shujuan Meng, Hecheng Zhou, Ziyang Feng, Zihao Xu, Ying Tang, Minghua Wu

**Affiliations:** ^1^Hunan Provincial Tumor Hospital, The Affiliated Tumor Hospital of Xiangya Medical School, Central South University, Changsha, China; ^2^Key Laboratory of Carcinogenesis and Cancer Invasion, Ministry of Education, Cancer Research Institute, School of Basic Medical Science, Central South University, Changsha, China; ^3^Key Laboratory of Carcinogenesis, Ministry of Health, Cancer Research Institute, School of Basic Medical Science, Central South University, Changsha, China

**Keywords:** circular RNA, epigenomics, neurodevelopment, m^6^A, non-coding RNA

## Abstract

Canonical epigenetic modifications, including DNA methylation, histone modification and chromatin remodeling, play a role in numerous life processes, particularly neurodevelopment. Epigenetics explains the development of cells in an organism with the same DNA sequence into different cell types with various functions. However, previous studies on epigenetics have only focused on the chromatin level. Recently, epigenetic modifications of RNA, which mainly include 6-methyladenosine (m^6^A), pseudouridine, 5-methylcytidine (m^5^C), inosine (I), 2′-*O*-ribosemethylation, and 1-methyladenosine (m^1^A), have gained increasing attention. Circular RNAs (circRNAs), which are a type of non-coding RNA without a 5′ cap or 3′ poly (A) tail, are abundantly found in the brain and might respond to and regulate synaptic function. Also, circRNAs have various functions, such as microRNA sponge, regulation of gene transcription and interaction with RNA binding protein. In addition, circRNAs are methylated by *N*^6^-methyladenosine (m^6^A). In this review, we discuss the crucial roles of epigenetic modifications of circRNAs, such as m^6^A, in the genesis and development of neurons and in synaptic function and plasticity. Thus, this type of changes in circRNAs might be a therapeutic target in central nervous system (CNS) disorders and could aid the diagnosis and treatment of these disorders.

## Introduction

Epigenomics, which refers to all the molecular pathways that modulate the expression of a genotype into a particular phenotype without any changes to the genome, plays an important role in the growth and development of mammals (Dupont et al., 2009). Research on canonical epigenetics has concentrated on DNA modifications and chromatin variations, and RNA epigenetic modifications, particularly those in non-coding RNAs, have recently garnered increasing attention. With the development of RNA deep sequencing technology and bioinformatics approaches, circRNAs have become increasing important among non-coding RNAs. Unlike linear RNAs, circRNAs have covalently closed loop structures without 5′ caps or 3′ poly-A tails due to back-splicing (Bolisetty and Graveley, 2013). Because of their stability (Suzuki and Tsukahara, 2014), evolutionary conservatism (Jeck et al., 2013) and high abundance (Gruner et al., 2016), circRNAs act as miRNA sponges (Hansen et al., 2013; Memczak et al., 2013), factors of RNA splicing (Ashwal-Fluss et al., 2014), and modulators of the expression of parental genes (Li Z. et al., 2015). circRNAs can also serve as biomarkers for numerous diseases (Meng et al., 2017). The latest studies have demonstrated that circRNAs can be methylated by m^6^A (Yang et al., 2017; Zhou et al., 2017), and its translation is enhanced by METTL3 and METTL14, and inhibited by FTO (Yang et al., 2017). Both circRNAs and m^6^A are involved in RNA processing and are related to neurodevelopment (Dominissini et al., 2012; Meyer et al., 2012; Rybak-Wolf et al., 2015). So, this review describes the effect of canonical epigenetics in neurodevelopment, summarizes the progress on RNA epigenetics and circRNAs, and suggests the relationship between neurodevelopment and circRNA epigenetics.

## Canonical Epigenetics and Neurodevelopment

Epigenetics is involved in many vital biological processes and plays an important role in the growth and development of organisms. Epigenetics explains how cells that carry the same genetic information differentiate into different cell types with various functions (Gapp et al., 2014). It is difficult to succeed in the treatment of neurological diseases, such as Parkinson’s disease, Alzheimer’s disease (AD), gliomas, and epilepsy. Thus, a study of the relationship between epigenetics and neurodevelopment contributes to our understanding of the occurrence and development of these diseases. The traditional epigenetics processes include DNA methylation, histone modification and chromatin remodeling. In this section, we demonstrate that DNA methylation, histone modification and chromatin remodeling play a role in neurodevelopment.

### DNA Methylation and Neurodevelopment

**FIGURE 1:**
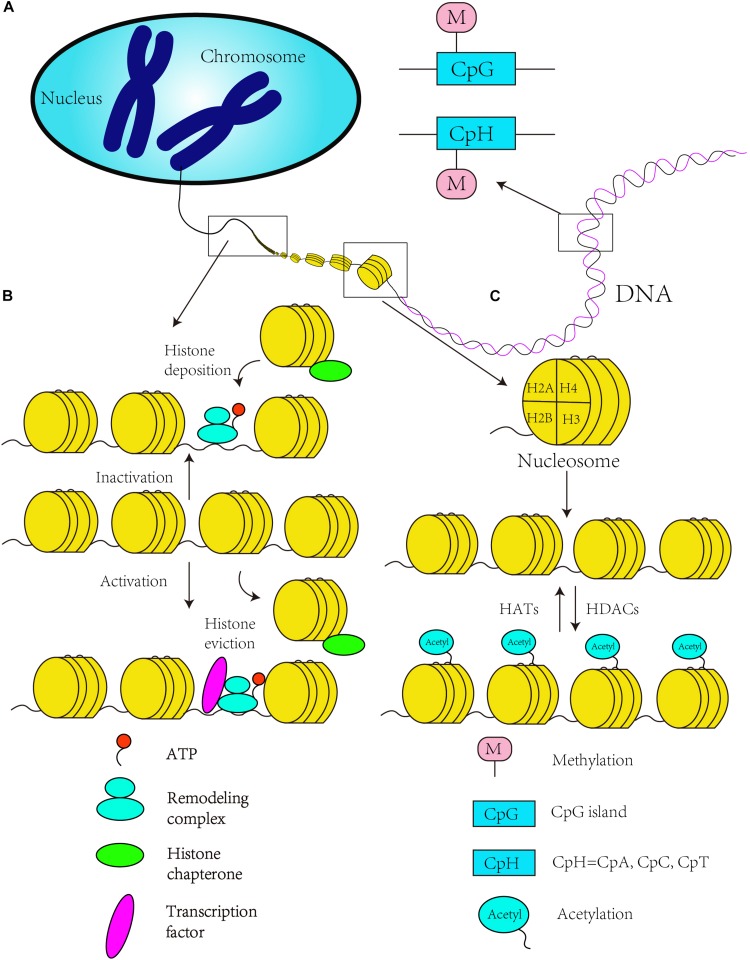
Canonical epigenetics mechanism. **(A)** DNA methylation in the CpG and CpH context. **(B)** Chromatin remodeling is ATP dependent. ATP-dependent remodeling complexes regulate DNA accessibility, and histone chaperones move and transfer histones on and off a locus. An open chromatin structure enables RNA polymerase II to catalyze the transcription when the histone is evicted, while chromatin state change to inactivity to inhibit gene transcription with the histone deposition of chromosome. **(C)** The acetylation of histone tails by histone acetyltransferases (HATs) establishes a more relaxed state in the chromatin that results in transcriptional activation (activated state). Histone deacetylation by HDACs reverses the activating chromatin state to a “inactive” chromatin state to inhibit gene transcription.

DNA methylation, as a covalent modification of genomic DNA, modifies gene expression and provides a mechanism for transmitting and perpetuating epigenetic information through DNA replication and cell division (Harman and Martin, 2019). Early development includes two stages of epigenetic programming: the first stage involves DNA demethylation or remethylation and the reprograming of histone PTMs in somatic cells, and the second stage guarantees and rebuilds parental imprints during germ cell development through DNA methylation (van Montfoort et al., 2012; Gapp et al., 2014). In addition, the neurodevelopment process also highly depends on DNA methylation (Gapp et al., 2014). Specifically, DNA methylation is observed at CpG islands in the mammalian genome, where it can modulate gene expression (Jones, 2012), through the addition of a methyl group to m^5^C molecules (Pulido Fontes et al., 2015). Brain DNA has one of the highest levels of m^5^Cs in human organs (Kriaucionis and Heintz, 2009; LaSalle et al., 2013), and DNA methylation is required for neuronal differentiation in mammals (Takizawa et al., 2001; Mohn et al., 2008). In addition, researchers have found that DNA methylation changes with LTP and m^5^C methyltransferase regulate synaptic plasticity in the hippocampus (Levenson et al., 2006). Some recent studies confirmed that in the presence of LTP, the methylation status of LTP genes undergoes widespread changes in the adult brain (Maag et al., 2017). These studies also showed that the methylation of Bdnf CpG islands is related to isoform switching from transcripts (Maag et al., 2017). Coincidentally, some researchers have identified that CpG island enriched for genes related to development and neurodifferentiation in schizophrenia patients, and widespread DNA methylation changes in schizophrenia-associated CpGs, were related to the transition from fetal brain cortex to postnatal development (Jaffe et al., 2016) (Figure 1A). Sleep deprivation can alter the cortical genome-wide distribution of DNA methylation, and these differences are enriched in gene pathways involving in the synapse formation and synaptic plasticity (Massart et al., 2014). In addition, some studies have found that gene-specific DNA methylation occurs in response to folic acid supplementation during pregnancy and is related to brain development and function (Caffrey et al., 2018).

### Histone Modification and Neurodevelopment

The basic structural unit of chromatin is the nucleosome, which is composed of one H3/H4 tetramer, two H2A and H2B dimers, and one H1 molecule. Histone modification refers to the set of covalent PTMs of histone proteins, and these modifications, which mainly include methylation, acetylation, phosphorylation, ubiquitination, sumoylation, and ADP-ribosylation (Berger, 2007), have been proven to be important in stem cell differentiation and neurodevelopment (Podobinska et al., 2017). For instance, class I and II HDACs, which contain two catalytic domains, act as the regulators of histone acetylation in mammals (Zhang et al., 2006; Podobinska et al., 2017). Many protein complexes that contain HDACs, such as SIN3/HDAC (Silverstein and Ekwall, 2005), the NuRD (Xue et al., 1998), co-repressor for element-1-silencing transcription factor (CoREST) (Shi et al., 2004) and the nuclear receptor co-repressor (N-CoR) (Jepsen et al., 2007), play important roles in neurodevelopment. These complexes not only catalyze the deacetylation of histones but are also associated with the activation of gene expression (Shimbo et al., 2013) or involved in the neural commitment and differentiation of stem cells (Podobinska et al., 2017). Learning and memory are vitally important processes in the growth and development of individual, and are necessary in brain development. Some animal experiments have shown that lacking HDAC2 or HDAC3 could improve learning (Guan et al., 2009; McQuown et al., 2011; Narayan and Dragunow, 2017), while loss of HDAC4 and HDAC5 has been shown to damage memory function (Kim et al., 2012; Sando, et al., 2012; Agis-Balboa et al., 2013), especially, HDAC4, which also participates in experience-dependent plasticity of synaptic (Agis-Balboa et al., 2013). Conversely, in the aged brain, HDACs and HATs, as transcription repressors to catalyze histone deacetylation, have been reported to be altered, and could be linked to age-related altered gene transcription (Barter and Foster, 2018). Some scientists have proven that the same isoforms of HDAC3 and HDAC4 were undetectable in the human AD prefrontal cortex compared to mouse models of AD, which had relatively high concentrations (Anderson et al., 2015). And comparison to the control cases, HDAC1 and HDAC2 were decreased but HDAC5 and HDAC6 were significantly increased in AD patient (Anderson et al., 2015), which implied that isoform selectivity of HDACs could be a target of therapy (Narayan and Dragunow, 2017). Although HDAC4 is undetectable in normal human brain, the expression of brain tumor tissue was increased (de Ruijter et al., 2003). Interestingly, deletion or mutation of HDAC4 results in reduced expression of *RAI1* can cause mental retardation, such as Smith–Magenis syndrome (Williams et al., 2010) (Figure 1C).

### Chromatin Remodeling and Neurodevelopment

Chromatin remodeling refers to dynamic modifications of the chromatin architecture that regulate transcription through displacement and rearrangement of the nucleosome. The process of chromatin remodeling is driven by ATP (Zaghlool et al., 2016), and chromatin remodeling complexes can be classified into four main classes: SWI/SNF, ISWI, INO80, and Mi2/CHD (Olave et al., 2002; Choi et al., 2015) (Figure 1B). A few recent studies have focused on chromatin remodeling and neurodevelopment. The Wnt signaling pathway is one of the most important pathways in embryonic development and axis patterning (Salinas, 2012), and some researchers have found that this signaling pathway can be repressed by one of the BRG1-associated factors in the ARID1B chromatin remodeling complex (also known as the SWI/SNF-A complex) (Vasileiou et al., 2015). This signaling pathway can be affected by a mutation in bromodomain adjacent to zinc finger domain protein 1A (BAZ1A), which encodes ATP-utilizing chromatin assembly and remodeling factor 1 (ACF1) (Zaghlool et al., 2016), and the mutation in BAZ1A also affects the development of proper synaptic functions (Zaghlool et al., 2016). Furthermore, chromatin remodeling might be influenced by persistent exposure to 6OH-BDE-47 (brominated diphenyl ether, BDE) and thereby affects downstream processes, such as synapse development and the overall functional maturity of neurons (Poston et al., 2018). Also, chromatin remodeling can be regulated by non-coding RNAs. For example, microRNA-9 and microRNA-124a could inhibit the expression of gene *BAF53a* (also known as ACTL6a), which is a component of SWI/SNF chromatin remodeling complexes, by corresponding to the recognition sites of 3′ untranslated region. Ultimately, neural progenitor proliferation was repressed (Yoo et al., 2009). As for the aging brain, chromatin remodeling driven by histone modifications is tightly related to the enzymes which can regulate the process of modifications added or removed (Harman and Martin, 2019). However, regulation and function of these enzymes is altered during brain aging leading to changes in the epigenome (Pal and Tyler, 2016). To some extent, these studies have demonstrated that chromatin remodeling plays a role in neurodevelopment and can affect embryonic development.

## Rna Modification

Epigenomics refers to stable and heritable changes in gene expression that do not alter the DNA sequence (Berger et al., 2009). However, epigenetic modifications occur not only in DNA but also in RNA, called the epitranscriptome, but the heritability of RNA modifications needs further study. Epitranscriptome includes more than 100 types of RNA modifications (Sun et al., 2016), and researchers have found that RNA modifications are abundant in tRNAs, rRNAs, and snRNAs but relatively rare in mRNAs (Lee et al., 2014; Sun et al., 2016). But in the last several years, technological advances improving our ability to identify mRNA modifications and recent studies of the cellular transcriptome have focused attention on epitranscription (Flamand and Meyer, 2019). Many of these modified transcripts in the brain are associated with autism and other neurodevelopmental disorders, and have implied that the epitranscriptome may impact the development and maturation of synapses (Washbourne, 2015; Flamand and Meyer, 2019). To a great extent, these modifications enrich the functions of RNA and genetic diversity (Maden, 1990; Wang X. et al., 2014; Zhang and Jia, 2016), and the common RNA modifications include pseudouridine (Ψ), m^6^A, 5-methylcytosine (m^5^C), m^7^G, *N*^1^-methyladenosine, and N_m_ (Sun et al., 2016; Zhang and Jia, 2016). This section summarizes the most common types of epigenetic modifications of RNA.

### *N*^6^-Methyladenosine (m^6^A)

*N*^6^-Methyladenosine, which refers to the methylation of position N^6^ of adenosine, is one of the most abundant modifications of mRNAs found in all eukaryotes. Early studies used mass spectrometry to detect this modification and revealed that the relative m^6^A content ranged from 0.1 to 0.4% (Wei et al., 1975), which corresponds to the modification of approximately three to five sites in each mRNA (Wei et al., 1975; Lee et al., 2014). The m^6^A modification, which is post-transcriptionally decoded by m^6^A methyltransferase, is a prevalent internal modification in eukaryotic mRNA (Wang X. et al., 2014), and always occurs in the consensus sequence RRACH (R = G or A; H = A, C or U) (Niu et al., 2013). To detect and analyze the location of m^6^A, researchers have developed a m^6^A-specific MeRIP-Seq approach and found that m^6^A is mainly concentrated in the 3′ UTRs of mRNAs, long internal exons and the stop codons (Meyer et al., 2012). The distribution of m^6^A in tissue-specific sites has also been investigated, and the results revealed that this modification is most abundantly found in the heart, brain and kidney (Meyer et al., 2012). Furthermore, the distribution of m^6^A is richer in the adult brain than in the fetal brain (Meyer et al., 2012). Coincidentally, Dominissini D et al. used an m^6^A-seq approach and found that the sites modified by m^6^A are highly conserved in humans and mice (Dominissini et al., 2012). Antibody-based crosslinking strategies have been developed in recent years to increase the resolution of m^6^A (Chen K. et al., 2015; Linder et al., 2015; Roundtree and He, 2016).

To more accurately describe the process of m^6^A, researchers have used the terms “writer,” “eraser,” and “readers,” and these terms are extensively used for many types of modifications, not just m^6^A methyltransferase. This modification is considered a “writer,” which uses the SAM cofactor as the methyl donor, and this cofactor is post-transcriptionally methylated at the N^6^ position of adenosine. m^6^A methyltransferase consists of METTL3, METTL14 and the regulatory subunit WTAP (Bokar et al., 1994; Liu et al., 2014; Ping et al., 2014). METTL14 has enzymatic activity (Liu et al., 2014), interacts with METTL3 and preferentially methylates the conserved GGACU and GGAUU sequences (Liu et al., 2014). Even though it does not have the activity of methyltransferase due to the lack of a catalytic center, WTAP can locate the methyltransferase complex to nuclear speckles by interacting with METTL3 and METTL14 (Ping et al., 2014). The knockdown of METTL3 causes changes in the splicing patterns and alternative polyadenylation, and influences RNA stability, transcriptional silencing, and translation (Dominissini et al., 2012; Schwartz et al., 2014; Ke et al., 2015; Meyer et al., 2015; Zhou et al., 2015; Lin et al., 2016; Patil et al., 2016; Wang X. et al., 2016; Pendleton et al., 2017). A recent study revealed a new mechanism of m^6^A: METTL16, a long unknown U6 snRNA methyltransferase able to control the SAM levels, which influence the level of m^6^A in most cells by regulating the expression of human MAT2A (Pendleton et al., 2017).

The discovery of m^6^A demethylating enzymes, named “erasers,” focused on FTO (Dina et al., 2007) and ALKBH5, which are proteins that belong to the Fe (II) and 2-oxoglutarate-dependent oxygenase superfamily (Jia et al., 2011; Zheng et al., 2013) and oxidize m^6^A through *N*^6^-hydroxymethyladenosine (hm^6^A) and *N*^6^-formyladenosine (f^6^A) intermediates (Fu et al., 2013). Recent studies have shown that FTO participates in many vital life processes, such as the regulation of dopaminergic signaling in the brain (Hess et al., 2013), the mRNA splicing of adipogenetic regulatory factors (Ben-Haim et al., 2015), adipogenesis (Zhao et al., 2014), and the enhancement of leukemic oncogene-mediated cell transformation and leukemogenesis (Li Z. et al., 2017). Both FTO and ALKBH5 are important in cells, and in HeLa cells, these demethylating enzymes also affect the processing, nuclear export and metabolism of mRNA (Zheng et al., 2013).

The effector proteins of m^6^A, which are called “readers,” include the YT521-B homology (YTH) family, which encodes five proteins, namely the YTH domain family (YTHDF) proteins 1, 2 and 3 and the YTH domain-containing (YTHDC) proteins 1 and 2 in mammals (Zhang et al., 2010; Li Z. et al., 2017). To date, four of these proteins have been shown to exhibit m^6^A selectivity *in vitro* and *in vivo* (Wang X. et al., 2014; Xu et al., 2014; Roundtree and He, 2016). YTHDF2 and YTHDC1 have a conserved hydrophobic binding pocket specific for m^6^A and participate in the process regulating the methylation and transcript fate of mRNA (Luo and Tong, 2014; Wang X. et al., 2014; Xu et al., 2014). In addition, the high-resolution mapping of transcription-binding sites has revealed that YTHDF1 and YTHDF2 prefer to bind to the GGACU conserved sequence motif in mRNA, which shows substantial overlap with sites of m^6^A methylation (Zhu et al., 2014; Wang et al., 2015; Roundtree and He, 2016).

*N*^6^-Methyladenosine plays a critical role in the development of an organism, and changes in the levels of m^6^A have an impact on many life processes, including tissue development, stem cell self-renewal (Wang Y. et al., 2014; Zhao et al., 2014) and differentiation (Geula et al., 2015). m^6^A can also control the heat shock response (Zhou et al., 2015), circadian clocks (Fustin et al., 2013), and processes associated with the fate and function of RNAs, such as the stability, splicing, transport, localization and translation of RNAs (Zheng et al., 2013; Wang X. et al., 2014; Wang Y. et al., 2014; Wang et al., 2015; Zhao et al., 2014; Meyer et al., 2015; Zhou et al., 2015), primary microRNA processing (Alarcon et al., 2015; Chen T. et al., 2015), and RNA-protein interactions (Dominissini et al., 2012; Meyer et al., 2012; Liu et al., 2015). Similarly, in neurodevelopment, m^6^A still has a critical role in reducing brain volume (Ho et al., 2010). In the developing cortex, m^6^A is abundant and controls the ample transcripts involved in neurogenesis and neuronal differentiation (Yoon et al., 2017; Flamand and Meyer, 2019). With the age growing, the m^6^A levels is increasing, especially in adulthood via controlling synaptic plasticity in the mature brain (Meyer et al., 2012). However, a considerable body of evidence indicates a relationship between m^6^A and diseases. In fact, it has been demonstrated that m^6^A is related to obesity, diabetes and cancer (Klungland and Dahl, 2014; Zelinski et al., 2014). Meanwhile, FTO also participate in regulation of learning and memory. For instance, decreasing the expression of *Fto* in the hippocampus causes the enhanced contextual fear memory and impaired LTP (Walters et al., 2017; Engel et al., 2018). Recent studies have shown that ALKBH5 and the depletion of m^6^A drive the formation of cancer stem cells (Jaffrey and Kharas, 2017). Coincidentally, a study conducted in 2017 revealed that m^6^A is relevant to the self-renewal and tumorigenesis of glioblastoma stem cells (Cui et al., 2017).

### Other RNA Modifications

Most studies on m^5^C have focused on DNA, and m^5^C is rare in RNA (Roundtree and He, 2016). However, researchers have discovered that m^5^C is enriched in the 3′-UTRs (Chhabra, 2015). 3-Methylcytidine (m^3^C) was first discovered in total RNA from *Saccharomyces cerevisiae* (Hall, 1963). The studies discovered that METTL2 and METTL6 have m^3^C modifications in specific tRNAs and that METTL8 only induces m^3^C modifications in mRNA in humans and mice (Xu et al., 2017). Some researchers successfully characterized RNA methylation in mixtures of either isomers of RNA or non-isomeric RNA forms and identified the RNA methylation modifications, including m^6^A, m^5^C, m^3^U, and m^5^U, by top-down mass spectrometry (Glasner et al., 2017).

Pseudouridine is also a relatively abundant type of RNA modification, and the relative amount of pseudouridine in RNA is in the range of 0.2–0.6% (Li X. et al., 2015). Pseudouridine formation involves two mechanisms: one is dependent on tRNA-pseudouridine synthase I and the other relies on a type of H/ACA box snoRNA (Charette and Gray, 2000; Ofengand, 2002). In rRNA, pseudouridine mainly appears in PTCs, decoding centers and the A-site finger region (ASF) (Jack et al., 2011). Thus, this modification might participate in the processing of rRNA, the assembly of ribosomes and the maintenance of advanced structures (Kiss et al., 2010). Studies have shown that in snRNA (U1, U2, U3, U4, U5, and U6), pseudouridine is highly conserved in different types of species (Yu et al., 2011). In 2011, some researchers showed that stop codons could be transformed into sense codons by pseudouridylation (Karijolich and Yu, 2011).

Inosine is a normal and essential post-transcriptional RNA modification introduced by specific deaminases (Alseth et al., 2014). In tRNA, this process is catalyzed by ADAT, whereas in mRNA and non-coding RNA, ADAR catalyze the process (Bass et al., 1997). In fact, A-to-I RNA editing plays a significant physiological role in neuronal function (Behm and Ohman, 2016). RNA encoding glioma-associated oncogene 1 (GLI1) is edited such that an arginine is changed to a glycine (R/G) in the protein. The GLI1 mRNA is highly edited, which induces an increase in the capacity of GLI1 to activate transcription by adenosine deamination in the normal cerebellum, but the process is obviously decreased in cell lines originating from cerebellar tumors (Shimokawa et al., 2013). Researchers have found that ADAR2 auto-editing is increased during mouse brain development and in rat primary cortical neuronal cultures, which suggests that ADAR2 activity is globally elevated (Hang et al., 2008; Behm and Ohman, 2016). In addition, modulated GluA2-4 R/G editing and alternative splicing generates AMPA receptors, which can adapt to differential rapid fast-synaptic transmission during development (Grosskreutz et al., 2003).

## Epigenetic Modifications of Circular RNAs

**FIGURE 2:**
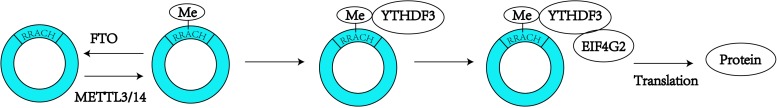
*N*^6^-Methyladenosine modification is always found to occur in the consensus sequence identified as: RRACH (R = G or A; H = A, C or U). M^6^A modification can promote the translation of the circRNAs.

In recent years, circRNAs have been one of the most frequently studied types of non-coding RNA. Due to their unique features, which are described above, circRNAs are known as miRNA sponges (Hansen et al., 2013; Memczak et al., 2013) and might also serve as potential biomarkers for a number of diseases, particularly cancers (Meng et al., 2017). Although numerous biological functions of circRNAs remain unknown, this field of research is being continuously explored. Some of the endogenous circRNAs identified to date have the capability of being translated into proteins through a process driven by the IRESs (Legnini et al., 2017). In early 2017, a group of researchers found that circRNAs can be widely methylated by m^6^A, as determined through the m^6^A immunoprecipitation of RNA samples treated with the RNase R exoribonuclease, and are efficiently translated through short sequences consisting of the m^6^A site as IRESs in human cells (Yang et al., 2017). The initiation of this m^6^A-mediated translation requires the eIF4G2 initiation factor and the YTHDF3 m^6^A reader, and the translation process is enhanced by METTL3/14 and inhibited by FTO (Yang et al., 2017). These researchers also inferred that proteins translated by circRNAs can be correlated with environmental stress (Yang et al., 2017). Coincidentally, other researchers designed a computational pipeline named AutoCirc to analyze the results from RNA and m^6^A immunoprecipitation and further demonstrated that m^6^A modifications are extensively observed in circRNAs. These researchers also showed that m^6^A circRNAs have highly cell-specific expression (Zhou et al., 2017), and revealed that circRNAs with m^6^A modifications also have long single exons. In addition, the researchers compared m^6^A circRNAs and m^6^A mRNAs and validated that the methylated exons in mRNAs are different from the exons that form m^6^A circRNAs (Zhou et al., 2017). Additionally, m^6^A circRNAs are related to mRNA stability through interaction with YTHDF1/YTHDF2 (Figure 2; Zhou et al., 2017). These studies expand the yield of RNA modifications and circRNAs, and more questions regarding circRNA modifications need to be expounded.

## CircRNAs and Neurodevelopment

In 2015, researchers used high-resolution *in situ* hybridization to verify that circRNAs are most abundantly found in the human brain (You et al., 2015), and some researchers have attempted to determine the reason for the enrichment of circRNAs in the brain (Chen and Schuman, 2016). It is well known that there are long introns (Jeck et al., 2013; Liang and Wilusz, 2014; Zhang et al., 2014) that flank circularized exons; thus, researchers have inferred that brain-specific genes might carry additional sequence features that can promote circRNA formation (Chen and Schuman, 2016). Although many functions of the circRNAs in brain remain unclear, it is undisputed that the circRNA levels in neurons are dynamically modulated. These studies illustrate that circRNAs play a vital role in neurodevelopment through these mechanism.

### MicroRNA Sponge and Interaction With RNA Binding Proteins

**FIGURE 3:**
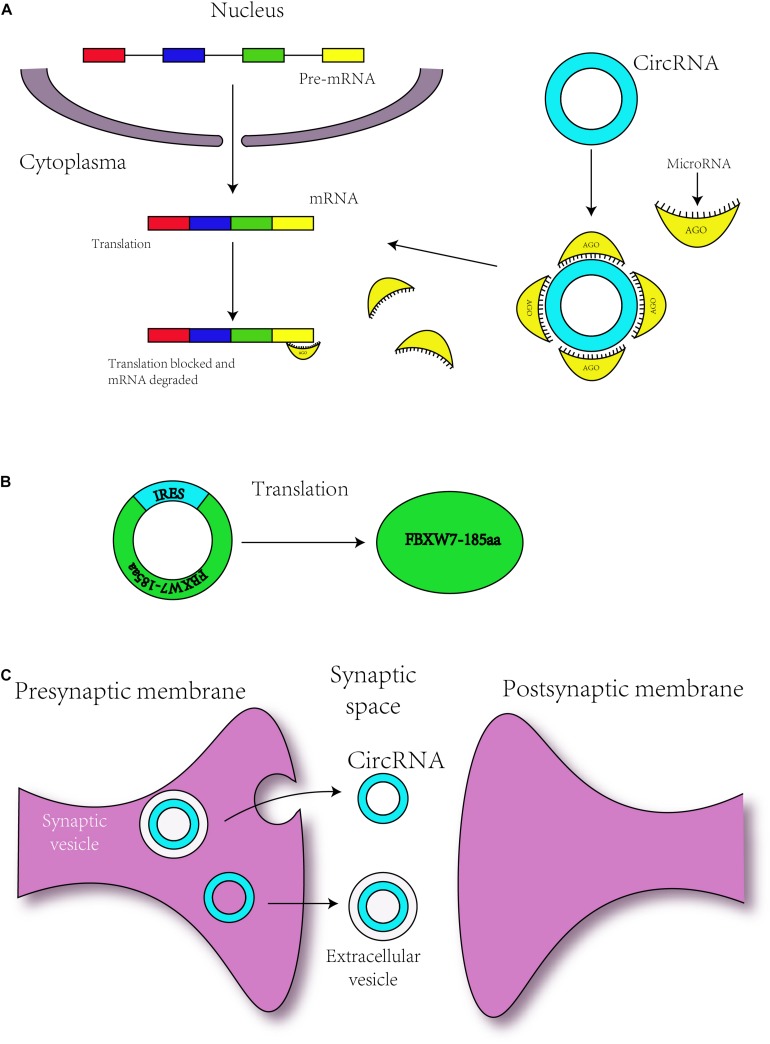
Circular RNAs are related to neurodevelopment through different mechanisms. **(A)** circRNAs regulate the translation of mRNAs by serving as miRNA sponges. **(B)** Some circRNAs with an IRES sequence can translate proteins, and this translation is driven by IRES. **(C)** circRNAs may transmit the information to environment by synaptosomes or extracellular vesicle such as exosomes.

Serving as a microRNA sponge was the first discovered function of circRNAs in 2013. The ciRS-7 contains more than 70 conserved binding sites for miRNA-7, and ciRS-7 can bind with Argonaute (AGO) protein (Figure 3A; Hansen et al., 2013). Additionally, the co-expression of ciRS-7 and miR-7 is distinctly high in neocortical and hippocampal neurons, which implies a high degree of endogenous interaction (Hansen et al., 2013). In 2017, researchers established the Cdr1as-loss mouse model by CRISPR/Cas9 (Piwecka and Glazar, 2017). Circ-Cdr1as reportedly binds to miR-7 and miR-671 (Hansen et al., 2011, 2013), and researchers have found that miR-7 and miR-671 are deregulated post-transcriptionally in the Cdr1as-knock-off brain. Furthermore, the expression of immediate early genes, such as Fos, which is the direct target of miR-7, is increased in the Cdr1as-loss mouse brain (Piwecka and Glazar, 2017). Cdr1as-knockout mice show defects in neuropsychiatric behaviors, which suggests that Cdr1as might be crucial for neuron-controlled behavior (Piwecka and Glazar, 2017). To ensure its interaction with RNA-binding proteins, Circ-Foxo3 is able to bind with many types of proteins, such as cell cycle-related proteins (Du et al., 2016), and thus, might participate in neuronal cell division in neurodevelopment.

### Regulation of Gene Transcription

Circular RNAs can also regulate gene transcription, but these effects are achieved via varied mechanisms. These circRNAs always exist in the nucleus. For example, in HeLa and HEK293 cells, circ-EIF3J and circ-PAIP2 are exon-intron circRNAs or EIciRNAs and interact with U1 snRNP to promote transcription of their parental genes (Li Z. et al., 2015). In 2013, researchers found a circular intronic RNA denoted ci-ankrd52 and found that this circRNA might affect the rate or efficiency of transcription as a positive regulator of Pol II transcription (Zhang et al., 2013). Therefore, these circRNAs might be related to transcription in neurons (Li Z. et al., 2015; van Rossum et al., 2016).

### Translation

As mentioned above, m^6^A can mediate the translation of circRNAs, but circRNAs can also be translated via other mechanisms. Some circRNAs contain the IRES responsible for driving translation, for instance, circ-ZNF609 and circMbl3 were found to translate proteins (Legnini et al., 2017; Pamudurti et al., 2017). In addition, in 2017, some researchers have found that circ-FBXW7 is abundantly expressed in the normal human brain and can encode a novel 21-kDa protein, the translation of which is driven by IRES (Figure 3B; Yang et al., 2018). The upregulation of this new protein can inhibit the proliferation and cell cycle acceleration of glioma cells (Yang et al., 2018). As a consequence, circRNAs might translate the proteins to regulate the process of neurodevelopment.

### Neurodevelopment and Synaptic Function

In 2015, some researchers have confirmed that circRNAs with biological functions are correlated with synaptic function and are significantly enriched in synapses, parts of the synapse, presynaptic active zones, presynaptic membranes and postsynaptic density (Figure 3C; You et al., 2015). circDscam, circKlhl2, circElavl3, circNlgn1, circGigyf2, circNbea, and circRmst are derived from synapse-related genes (You et al., 2015), indicating a relationship between circRNAs and synaptic function. In addition, highly expressed circRNAs are derived from synaptic genes, such as Dscam and Homer1, and might participate in Wnt signaling, axon guidance and TGF-β signaling (Veno et al., 2015; You et al., 2015). During hippocampal and Drosophila brain development, the expression of circRNAs is developmentally upregulated (Westholm et al., 2014; You et al., 2015) and can be regulated by neural plasticity (You et al., 2015). These findings revealed the role of circRNAs in brain development. Other researchers have found that the expression level of circRNAs shows differences among various brain areas (Rybak-Wolf et al., 2015).

## The Potential Roles of circRNA Epigenetic Modifications in Neurodevelopment

As mentioned above, m^6^A is one of the most abundant methylation patterns in mRNA and is also present in circRNAs (Yang et al., 2017; Zhou et al., 2017). In addition, FTO, as m^6^A demethylating enzymes, were found to display dynamic expression in postnatal neurodevelopment (Li L. et al., 2017). FTO deficiency not only results in a decreased brain size and a reduced body weight but also leads to impairments in learning and memory (Li L. et al., 2017). Further studies have illustrated that m^6^A is indispensable for the regulation of RNA fate and function, which are central to differentiation and growth (Geula et al., 2015). In addition, the majority of circRNAs are upregulated during the development of the Drosophila brain, but some circRNAs are downregulated (You et al., 2015; Zhou et al., 2017). By serving as miRNA sponges, circRNAs are involved in the regulation of RNA processing, such as alternative splicing, pre-RNA splicing and RNA editing (Hansen et al., 2013; Starke et al., 2015; van Rossum et al., 2016). Their expression level is regulated by synaptic plasticity during neurodevelopment. As a result, we hypothesized various mechanisms through which epigenetic circRNAs affect neurodevelopment. First, the epigenetic modification of circRNAs might occupy miRNA sites, which can prevent miRNA-mRNA binding. Second, during neurodevelopment, epigenetic circRNAs might transmit information to the microenvironment by exosomes. Exosomes were first found in 1983 as a type of 50-nm vesicles (Harding et al., 2013), and play a significant role in intracellular and extracellular communication. Some studies have demonstrated that pre-miRNAs with Dicer, AGO2, and *trans-*activation response RNA binding protein (TRBP) are present in exosomes of breast cancer cells (Melo et al., 2014). Therefore, circRNAs with epigenomic changes might regulate the biogenesis and contents of exosomes to participate in the early formation and plasticity of synapses. Third, some circRNAs can translate the protein, and these proteins might play a role in RNA processing. Thus, we can infer that epigenetic modifications of circRNAs, such as m^6^A, might play a vital role in genesis and neurodevelopment by impacting the alternative splicing of RNAs and in synaptic function and plasticity by influencing RNA processing. In addition, circRNAs participate in many CNS diseases, such as GBM (Zhu et al., 2017), CNS lymphoma (Baraniskin et al., 2016), cerebral ischemia (Ouyang et al., 2013), stroke (Ouyang et al., 2013), Alzheimer’s disease (Wu et al., 2013), Huntington’s disease (Wu et al., 2013), and Parkinson’s disease (Wu et al., 2013). This finding indicates that circRNAs might serve as biomarkers of CNS disorders (Qu et al., 2015; Lu and Xu, 2016). Therefore, in view of these data, changes in the epigenetic modifications of circRNAs might influence RNA stability and lead to neuronal disorders. circRNAs might be a therapeutic target of CNS disorders and can potentially aid the diagnosis of various diseases.

## Conclusion

In conclusion, although changes in the epigenetic modifications of circRNAs could exert an effect on neurodevelopment and CNS diseases, considerable studies are needed to confirm this finding. It is thus important to identify the changes in circRNA epigenetic modifications in neurodevelopment and to find the mechanisms of these modifications, which could reveal the roles of circRNAs in CNS diseases. These studies might aid the diagnosis and treatment of CNS diseases.

## Author Contributions

ZF, ZX, and YT collected the related manuscript. SM drafted and revised the manuscript. HZ drew the figures. MW participated in the design of the review and helped to draft and revised the manuscript. All authors read and approved the final manuscript.

## Conflict of Interest Statement

The authors declare that the research was conducted in the absence of any commercial or financial relationships that could be construed as a potential conflict of interest.

## Abbreviations

ADAR: adenosine deaminases acting on RNA
ADAT: adenosine deaminases acting on tRNA
ALKBH5: AlkB homolog 5
ARID1B: AT-rich interactive domain-containing protein 1B
Bdnf: brain-derived neurotrophic factor
CHD: chromodomain helicase DNA-binding protein
circRNA: circular RNA
ciRS-7: circular RNA sponge for miR-7
CNS: central nervous system
CoREST: corepressor for element-1-silencing transcription factor
DSCAM: Down Syndrome cell adhesion molecule
FTO: fat mass-and-obesity-associated protein
GBM: glioblastoma multiforme
HATs: histone acetyl-transferases
HDACs: histone deacetylases
IRESs: internal ribosome entry sites
ISWI: Imitation SWItch
LTP: long-term potentiation
m^1^A: 1-methyladenosine
m^5^C: 5-methylcytidine
m^5^C: carbon 5 of cytosine
m^6^A: *N*^6^-methyladenosine
m^7^G: *N*^7^-methylguanosine
MeRIP-Seq: methylated RNA immunoprecipitation with next-generation sequencing
METTL14: methyltransferase-like 14
METTL3: methyltransferase-like 3
miRNA: microRNA
N-CoR: nuclear receptor corepressor
Nm: 2 ′ -*O*-methylation
NuRD: nucleosome remodeling and deacetylase complex of histone acetylation
PTC: peptidyl-transferase center
PTMs: post-translational modifications
SAM: S-adenosyl methionine
SWI/SNF: SWItch/Sucrose Non-Fermentable
TRBP: transactivation response RNA-binding protein
UTRs: untranslated regions
WTAP: Wilms’tumor 1-associating protein
YTHDC: YTH domain-containing protein
YTHDF: YTH domain-containing family.
